# On the Multiuser Diversity of Relay-Aided Downlink Systems Using Reduced Feedback

**DOI:** 10.1155/2014/857540

**Published:** 2014-11-10

**Authors:** Yong-Up Jang, Won-Yong Shin

**Affiliations:** ^1^Agency for Defense Development, Daejeon 305-600, Republic of Korea; ^2^Department of Computer Science and Engineering, Dankook University, Yongin 448-701, Republic of Korea

## Abstract

We introduce an efficient multiuser scheduling method using amplify-and-forward relaying in relay-aided downlink systems, consisting of one base station (BS), one relay station, and multiple mobile stations (MSs). In our scheme, the BS opportunistically selects both the transmission mode, that is, either one- or two-hop transmission, and the desired user (i.e., the desired MS). Closed-form expressions for the average achievable rates are derived for the two transmission modes with multiuser scheduling, and its asymptotic solutions are also analyzed in the limit of large number of MSs. Based on the analysis, we propose a *feedback-efficient* two-step multiuser scheduling algorithm: the transmission mode selection followed by the user selection that only needs a partial feedback for instantaneous signal-to-noise ratios (SNRs) to the BS. We also analyze the *average SNR* condition such that the multiuser diversity gain is fully exploited for two-hop transmission. The proposed two-step scheduling algorithm exhibits the quite comparable achievable rates to those of the optimal one using full feedback information, while its required feedback information is reduced by half of the optimal one.

## 1. Introduction

Cooperative relaying transmission techniques have widely been studied owing to the advantages of both enhancing the end-to-end link quality in terms of system capacity and extending the coverage [1]. One of the simplest relay protocols is the use of a half-duplex two-hop relaying which assists the communication between a base station (BS) and a mobile station (MS) that is geographically located far apart from the BS [2]. There have been intensive prior studies on considering half-duplex two-hop relay cooperation strategies, which include a variety of novel techniques such as distributed space-time coding [3, 4] and relay station (RS) selection [5], where one BS, one MS, and multiple RSs are deployed in wireless cooperative systems. However, one disadvantage of such half-du plex two-hop relaying schemes is that resources, for example, time and frequency, are required twice compared to those for one-hop transmission with no relaying. Thus, the half-duplex two-hop relay schemes in [3–5] induce the loss of system capacity up to the pre-log factor 1/2 [6] and cannot always guarantee a better performance on the system capacity compared to the one-hop transmission case. (To eliminate the pre-log factor 1/2, a full-duplex two-hop relaying method was proposed by utilizing additional antennas at a relay [7]. In this paper, we take only into account a half-duplex relaying scenario for easy implementation.) Hence, a proper scheduling algorithm, which selects the transmission mode, that is, either one- or two-hop transmission, according to link conditions, should be adopted to maximize the system capacity.

In wireless systems with many users, a multiuser diversity gain can be exploited to further improve the system throughput by utilizing the usefulness of fading since there are a large number of MSs in multiuser environments: opportunistic scheduling [8], opportunistic beamforming [9], and random beamforming [10] in single-cell downlink systems. Such opportunism has also been studied in multicell uplink networks through a distributed opportunistic scheduling [11, 12]. Moreover, the concept of opportunistic interference alignment has been introduced in [13–17] for cellular uplink and downlink networks, also known as the interfering multiple-access channel and interfering broadcast channel, respectively, where user scheduling is incorporated into the classical interference alignment framework by opportunistically selecting certain users in each cell. Scenarios obtaining the multiuser diversity have also been studied in cooperative networks by applying an opportunistic two-hop decode-and-forward (DF) relaying [18, 19] and an opportunistic multiuser two-way amplify-and-forward (AF) relaying [20], in ad hoc networks by introducing an opportunistic routing [21] and in cognitive radio networks with opportunistic scheduling [22].

In this paper, we focus on a relay-aided downlink system, commonly referred to as one of practical cooperative communication systems, which consists of one BS, one RS, and *N* MSs. As a combination of the traditional broadcast channel and relay channel, it matches several scenarios such as the downlink cellular environment with relay and wireless network with backbone nodes. To show the fundamental limits of such a relay-aided downlink system, in the literature [23–27], there are a lot of studies on characterizing the achievable rates for the channel where one BS transmits data packets to* multiple* MSs with the help of one RS (termed the broadcast relay channel). The schemes in [23–27] use either DF relaying [23–27] or compress-and-forward relaying [23, 26]. However, so as to obtain relatively high sum-rates approaching the system capacity, they were designed based on the use of rather complex nonlinear processing (i.e., a superposition coding at the BS and a successive or joint decoding at the receiver sides). On the other hand, under the relay-aided downlink system with one RS support, when the BS is assumed to serve* only one* MS, one may design a practical achievable scheme along with user scheduling, which requires no complex multiuser detection and thus yields an easier implementation. In this case, the best scheduling method is for the BS to simultaneously select both the transmission mode and the desired user (i.e., the desired MS) among all users, based on instantaneous signal-to-noise ratios (SNRs) of one- and two-hop links, including the channel conditions, thereby requiring the huge amount of feedback information.

As an alternative approach, in the same system model with one MS selection, we propose a two-step multiuser scheduling method, using two-hop amplify-and-forward (AF) relaying, based on efficiently* reduced feedback* information. In our scheme, the BS opportunistically selects both the transmission mode, that is, either one- or two-hop transmission, and the desired user (MS). First, the BS selects the transmission mode where a higher average achievable rate between two transmission modes with multiuser scheduling is achieved. Next, the desired user out of *N* MSs is opportunistically selected, based on partial feedback information at the BS, which includes instantaneous SNRs of all of either the one- or two-hop links. To construct a scheduling criterion, we derive a closed-form expression for the average achievable rates of two transmission modes with multiuser scheduling. Its asymptotic solutions are also analyzed in the limit of large *N*. From our analysis, the following interesting results are made: as *N* increases, the average achievable rate for two-hop transmission is either upperbounded by a constant or unbounded due to the multiuser diversity gain—the link condition such that the multiuser diversity gain is fully exploited is shown as a function of the* average SNRs* and *N*. In addition, numerical evaluation is performed via computer simulations to verify the performance of the proposed scheduling algorithm. We conclude that the proposed two-step scheduling method exhibits the quite comparable achievable rates to those of the optimal one using full feedback information, while its required feedback information is fairly reduced by half of the optimal one.

The organization of this paper is as follows.  Section 2 describes the system and channel models. In Section 3, the proposed two-step multiuser scheduling algorithm is shown. The average achievable rates of our multiuser scheduling are analyzed in Section 4.  Section 5 presents numerical evaluation via computer simulations. Finally, Section 6 summarizes this paper with some concluding remarks.

Throughout the paper, *E*[·] and *C* denote the expectation and the field of complex numbers, respectively. *CN*(0, *x*) represents a zero-mean circularly symmetric complex Gaussian distribution with variance *x*. Unless otherwise stated, all logarithms are assumed to be to the base 2.

## 2. System and Channel Models

**Figure 1:**
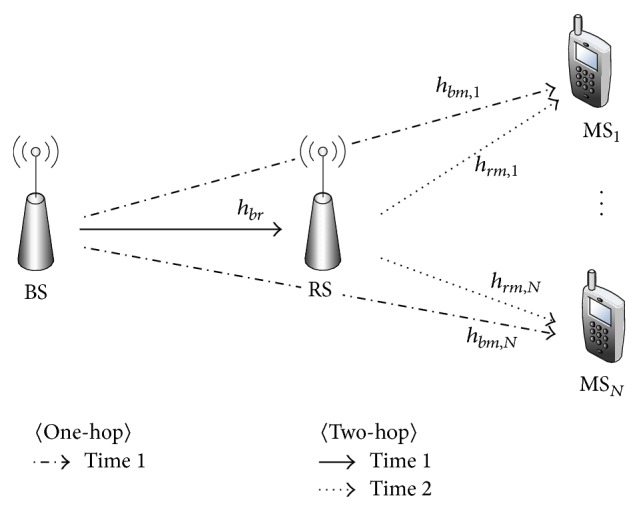
The relay-aided downlink system with one BS, one RS, and *N* MSs.

As illustrated in Figure 1, we consider the relay-aided downlink system, consisting of one BS, one RS, and *N* MSs. The channel model deals with the problem that one BS communicates with one selected MSs with or without the help of one RS. Hence, we do not assume that the BS sends different data packets to multiple MSs simultaneously since it requires the use of sophisticated multiuser detection schemes at the receivers.

For one-hop transmission, we perform a direct transmission from the BS to a certain selected MS. Then, the received signal at the *n*th MS is given by

(1) $$\begin{array}{c} y_{1,n}=h_{bm,n}\sqrt{P_{b}}x+w_{m,n},\;n=1,\ldots ,N, \end{array}$$

where *h*_*bm*,*n*_ ∈ *C* is the complex channel coefficient between the BS and the *n*th MS, *P*_*b*_ is the transmit power at the BS, *x* is the transmit signal, and *w*_*m*,*n*_ denotes the complex additive white Gaussian noise (AWGN) at the *n*th MS, which follows *CN*(0, *σ*_*m*_^2^).

For two-hop transmission, communication is performed from the BS to one MS through the RS. We assume that the RS operates in simple half-duplex, that is, the RS is not assumed to receive and transmit data packets simultaneously, and AF mode. Then, at the first time slot, the BS transmits its data to the RS, and at the second time slot, the RS amplifies and forwards the received data to the corresponding MS. For simplicity, we do not consider a direct path from the BS to MSs for two-hop transmission since two-hop transmission is required when the links between the BS and MSs are relatively poor. The received signal at the *n*th MS is then given by

(2) $$\begin{array}{c} y_{2,n}=h_{rm,n}g\left(h_{br}\sqrt{P_{b}}x+w_{r}\right)+w_{m,n},\;n=1,\ldots ,N, \end{array}$$

where *h*_*rm*,*n*_ ∈ *C* and *h*_*br*_ ∈ *C* are the complex channel coefficients between the RS and the *n*th MS and between the BS and the RS, respectively, *g* is the amplification factor at the RS, and *w*_*r*_ denotes the complex AWGN at the RS, following the distribution *CN*(0, *σ*_*r*_^2^). The channel coefficients *h*_*bm*,*n*_, *h*_*rm*,*n*_  (*n* = 1,…, *N*), and *h*_*br*_ are independent and identically distributed (i.i.d.) and are frequency-flat fading, where all the distributions are assumed to be *CN*(0,1). In our model, the amplification factor *g* at the RS is represented as [2]

(3) $$\begin{array}{c} g=\sqrt{\frac{P_{r}}{{\left|h_{br}\right|}^{2}P_{b}+\sigma_{r}^{2}}}, \end{array}$$

where *P*_*r*_ is the transmit power at the RS.

## 3. Proposed Two-Step Multiuser Scheduling

In this section, we propose a multiuser scheduling method that efficiently reduces the amount of feedback information for instantaneous SNRs from the MSs to the BS. The BS decides both the transmission mode (i.e., either one- or two-hop transmission) and the desired MS based on a scheduling criterion.

Let *R*_*i*_ denote the average achievable rate for *i*-hop transmission (*i* = 1,2) when an MS with the maximum instantaneous SNR is selected among *N* MSs. Suppose that all links between the BS and the MSs experience the same* average SNR*. All links between the RS and the MSs are also assumed to experience the same average SNR (this is a reasonable assumption since in the typical relay-aided cellular setup, an RS is located inbetween the BS and the cell-edge MSs). Let ${\bar{\gamma }}_{bm}$, ${\bar{\gamma }}_{rm}$, and ${\bar{\gamma }}_{br}$ denote the average SNRs of the BS-MS, RS-MS, and BS-RS links, respectively. Then, it follows that ${\bar{\gamma }}_{bm}=P_{b}/\sigma_{m}^{2}$, ${\bar{\gamma }}_{rm}=P_{r}/\sigma_{m}^{2}$, and ${\bar{\gamma }}_{br}=P_{b}/\sigma_{r}^{2}$. In this case, the rate *R*_*i*_ is expressed as a function of the average SNRs (i.e., ${\bar{\gamma }}_{bm}$, ${\bar{\gamma }}_{rm}$, and ${\bar{\gamma }}_{br}$) and the number of MSs, *N*, which will be analyzed in Section 4. Our scheduling algorithm consists of the following two steps.

**Step 1 (transmission mode selection).**

The transmission mode $\hat{i}\;$ is chosen by

(4) $$\begin{array}{c} \hat{i}=\underset{i\in \{1,2\}}{\text{argmax}}\left\{R_{i}\right\}. \end{array}$$

Note that the decision is made based on the average achievable rates *R*_1_ and *R*_2_, which can be numerically computed by using their closed-from expressions, depending on parameters ${\bar{\gamma }}_{bm}$, ${\bar{\gamma }}_{rm}$, ${\bar{\gamma }}_{br}$, and *N* (we remark that a closed-form expression for the rates *R*_1_ and *R*_2_ is derived in Section 4). Then, the mode $\hat{i}$ with a higher rate is selected.

**Step 2 (user selection).**

The BS requests the instantaneous SNR of the corresponding link to all the MSs. For one-hop transmission, the instantaneous SNRs of the BS-MS links given by using (1),

(5) $$\begin{array}{c} \frac{P_{b}{\left|h_{bm,n}\right|}^{2}}{\sigma_{m}^{2}}={\bar{\gamma }}_{bm}{\left|h_{bm,n}\right|}^{2}, \end{array}$$

for *n* = 1,…, *N*, should be fed back to the BS. For two-hop transmission, the BS only needs the instantaneous BS-RS-MS SNRs given by using (2),

(6) $$\begin{array}{c} \frac{g^{2}{\left|h_{rm,n}\right|}^{2}P_{b}{\left|h_{br}\right|}^{2}}{g^{2}{\left|h_{rm,n}\right|}^{2}\sigma_{r}^{2}+\sigma_{m}^{2}}=\frac{{\bar{\gamma }}_{br}{\left|h_{br}\right|}^{2}{\bar{\gamma }}_{rm}{\left|h_{rm,n}\right|}^{2}}{{\bar{\gamma }}_{br}{\left|h_{br}\right|}^{2}+{\bar{\gamma }}_{rm}{\left|h_{rm,n}\right|}^{2}+1}, \end{array}$$

for *n* = 1,…, *N*. Based on feedback information, the BS finally selects one MS which has the maximum instantaneous SNR of the corresponding link.

For comparison, the optimal multiuser scheduling, based on full feedback of the instantaneous SNRs, is also considered. In this case, both the transmission mode and the desired user are selected simultaneously in the sense of maximizing the instantaneous achievable rate for a given channel realization. In Section 5, it will be shown that the proposed two-step scheduling algorithm always shows higher average achievable rate than that of either one- or two-hop transmission, which is rather obvious, with the same amount of feedback information; it exhibits quite comparable performance on the achievable rates to those of the optimal one for which the amount of required feedback information is twofold.

## 4. The Analysis of Achievable Rates

In this section, the average achievable rates of both one- and two-hop transmissions with the proposed multiuser scheduling are analyzed. A closed-form expression for the average achievable rates is first derived, and its asymptotic behavior is then shown in the limit of large number of *N*.

### 4.1. One-Hop Transmission

From (5), the average achievable rate *R*_1_ for one-hop transmission is given by

(7) $$\begin{array}{c} R_{1}=E\left[\log\left(1+\text{SN}{\text{R}}_{1,\max}\right)\right], \end{array}$$

where $\text{SN}{\text{R}}_{1,\max}=\max_{n=1,\ldots ,N}\left\{{\bar{\gamma }}_{bm}|h_{bm,n}{|}^{2}\right\}$. In the following lemma, we derive a closed-form expression for (7).

**Lemma 1 .**

Suppose that the one-hop transmission with our multiuser scheduling is used. Then, the average achievable rate *R*_1_ is derived as

(8) $$\begin{array}{c} R_{1}=\frac{N}{\ln2}\overset{N-1}{\underset{n=0}{\sum }}{\left(-1\right)}^{n}\left(\begin{array}{c} N-1\\ n \end{array}\right)\frac{e^{\left(n+1\right)/{\bar{\gamma }}_{bm}}}{n+1}E_{1}\left(\frac{n+1}{{\bar{\gamma }}_{bm}}\right), \end{array}$$

where ${\bar{\gamma }}_{bm}=P_{b}/\sigma_{m}^{2}$ and *E*_1_(*x*) = ∫_*x*_^*∞*^(*e* ^−*t*^/*t*)*dt* is the exponential integral function.

**Proof**

The proof essentially follows the derivation of the selection combining scheme in [28].

Note that *R*_1_ is expressed as a function of both the average SNR of the BS-MS link, ${\bar{\gamma }}_{bm}$, and the number of MSs, *N*. For large *N*, the average achievable rate *R*_1_ in (8) is asymptotically given by

(9) $$\begin{array}{c} R_{1}\approx \log\left(1+{\bar{\gamma }}_{bm}\ln N\right), \end{array}$$

with high probability, which scales as log⁡⁡log⁡⁡*N* [9]. Hence, the multiuser diversity gain can be fully exploited for any average SNRs, that is, link conditions.

### 4.2. Two-Hop Transmission

We first show the maximum instantaneous SNR, termed SNR_2,max⁡_, for two-hop transmission with our multiuser scheduling. Using (6), we obtain SNR_2,max⁡_ as follows:

(10) $$\begin{array}{c} \text{SN}{\text{R}}_{2,\max}=\underset{n=1,\ldots ,N}{\max}\left\{\frac{\gamma_{br}\gamma_{rm,n}}{\gamma_{br}+\gamma_{rm,n}+1}\right\}, \end{array}$$

where $\gamma_{br}={\bar{\gamma }}_{br}|h_{br}{|}^{2}$ and $\gamma_{rm,n}={\bar{\gamma }}_{rm}|h_{rm,n}{|}^{2}$. The main characteristic for the right-hand-side of (10) is shown in Lemma 2.

**Lemma 2 .**

The function

(11) $$\begin{array}{c} f\left(\gamma_{rm,n}\right)=\frac{\gamma_{br}\gamma_{rm,n}}{\gamma_{br}+\gamma_{rm,n}+1} \end{array}$$

is monotonically increasing with respect to *γ*_*rm*,*n*_ for given *γ*_*br*_ > 0.

**Proof**

The first derivative of *f*(*γ*_*rm*,*n*_) with respect to *γ*_*rm*,*n*_ is always greater than 0 since

(12) $$\begin{array}{c} \frac{\partial f\left(\gamma_{rm,n}\right)}{\gamma_{rm,n}}=\frac{\gamma_{br}\left(\gamma_{br}+1\right)}{{\left(\gamma_{br}+\gamma_{rm,n}+1\right)}^{2}}>0, \end{array}$$

which completes the proof.

Thus, the average achievable rate *R*_2_ for two-hop transmission is given by

(13) R2=E12log⁡1+SNR2,max⁡=E12log⁡1+γbrmax⁡n=1,…,N⁡γrm,nγbr+max⁡n=1,…,N⁡γrm,n+1,

where the second equality comes from Lemma 2. As our first main result, a closed-form expression for (13) is derived in Theorem 3.

**Theorem 3 .**

Suppose that the two-hop transmission with our multiuser scheduling is used. Then, the average achievable rate *R*_2_ is written as

(14a) R2=N2ln⁡2γ¯brγ¯rm∑n=0N−1−1nN−1n ×1γ¯bre1/γ¯brE11γ¯br−n+1γ¯rmen+1/γ¯rmE1n+1γ¯rm ×n+1γ¯brγ¯rm1γ¯br−n+1γ¯rm−1 if  1γ¯br≠n+1γ¯rm

(14b) R2=N2ln⁡2γ¯brγ¯rm∑n=0N−1−1nN−1n ×γ¯br2−1+1γ¯br+1e1/γ¯brE11γ¯brif  1γ¯br=n+1γ¯rm,

where ${\bar{\gamma }}_{br}=P_{b}/\sigma_{r}^{2}$, ${\bar{\gamma }}_{rm}=P_{r}/\sigma_{m}^{2}$, and *E*_1_(*x*) = ∫_*x*_^*∞*^(*e* ^−*t*^/*t*)*dt* is the exponential integral function.

**Proof**

The probability density function (PDF) *p*_*γ*_*br*__(*x*) of the random variable *γ*_*br*_ is exponentially distributed and thus is given by

(15) $$\begin{array}{c} p_{\gamma_{br}}\left(x\right)=\frac{1}{{\bar{\gamma }}_{br}}e^{-\left(x/{\bar{\gamma }}_{br}\right)}. \end{array}$$

The PDF *p*_*γ*_*rm*,max⁡__(*y*) of the random variable *γ*_*rm*,max⁡_≜max⁡_*n*_{*γ*_*rm*,*n*_} is given by

(16) pγrm,max⁡y=Nγ¯rme−y/γ¯rm1−e−y/γ¯rmN−1=Nγ¯rm∑n=0N−1−1nN−1ne−n+1y/γ¯rm,

where the first and second equalities hold due to the order statistics [29] of an exponential random variable and the binomial theorem, respectively. Using (15) and (16) and then taking the double integral of the logarithmic term in (13) with respect to *x* and *y*, we have

(17) R2=N2ln⁡2γ¯brγ¯rm∑n=0N−1−1nN−1n ×∫x=0∞∫y=0∞Je−x/γ¯bre−n+1y/γ¯rmdxdy,︸Q

where *J* = ln⁡(1 + *x*) − ln⁡⁡(1 + (*x*/(1 + *y*))). Using (4.337.2) in [30] and the equality *E*_1_(*x*) = −*E*_*i*_(−*x*)≜−∫_−*x*_^*∞*^(*e* ^−*t*^/*t*)*dt*, the term *Q* in (17) is rewritten as

(18) Q=γ¯br∫y=0∞e1/γ¯brE11γ¯br−e1+y/γ¯brE11+yγ¯br     ×e−((n+1)y/γ¯rm)dy.

When $1/{\bar{\gamma }}_{br}\neq \left(n+1\right)/{\bar{\gamma }}_{rm}$, we obtain (14a) by applying the integral by parts to (18). When $1/{\bar{\gamma }}_{br}=\left(n+1\right)/{\bar{\gamma }}_{rm}$, we obtain (14b) by applying the integral by parts to (18) and using (6.221) in [30]. This completes the proof of this theorem.

In addition, we examine the asymptotic behavior of the average achievable rate *R*_2_ in (14a) and (14b) for large *N*. Unlike the asymptotic result for one-hop transmission, it is shown that full multiuser diversity gain is not always guaranteed for two-hop transmission case. We establish Theorem 4, which shows the link condition where the multiuser diversity gain is fully exploited in an asymptotic manner.

**Theorem 4 .**

Suppose that the two-hop transmission with our multiuser scheduling is used. When the number of MSs, *N*, is large and the average SNR of the RS-MS link, ${\bar{\gamma }}_{rm}$, does not scale with *N*, the average achievable rate *R*_2_ in (14a) and (14b) is asymptotically derived as follows:

(19a) $$\begin{array}{c} R_{2}\approx \frac{e^{1/{\bar{\gamma }}_{br}}E_{1}\left(1/{\bar{\gamma }}_{br}\right)}{2\ln2}\;if\;\;{\bar{\gamma }}_{br}=o\left(\ln N\right) \end{array}$$

(19b) $$\begin{array}{c} R_{2}\approx \frac{1}{2}\log\left(1+{\beta \bar{\gamma }}_{rm}\ln N\right)\;if\;\;{\bar{\gamma }}_{br}=\Omega \left(\ln N\right), \end{array}$$

with high probability, where ${\bar{\gamma }}_{br}=P_{b}/\sigma_{r}^{2}$, ${\bar{\gamma }}_{rm}=P_{r}/\sigma_{m}^{2}$, and *E*_1_(*x*) = ∫_*x*_^*∞*^(*e* ^−*t*^/*t*)*dt* is the exponential integral function. Here, *β* = 1 if ${\bar{\gamma }}_{br}=\omega (\ln N)$, and 0 < *β* < 1 if  ${\bar{\gamma }}_{br}=C_{0}\ln N$ for some constant *C*_0_ > 0. Note we use the following notation: (i) *f*(*x*) = *O*(*g*(*x*)) means that there exist constants *C* and *c* such that *f*(*x*) ≤ *Cg*(*x*) for all *x* > *c*; (ii) *f*(*x*) = *o*(*g*(*x*)) means lim⁡_*x*→*∞*_⁡(*f*(*x*)/*g*(*x*)) = 0; (iii) *f*(*x*) = *Ω*(*g*(*x*)) if *g*(*x*) = *O*(*f*(*x*)); and (iv) *f*(*x*) = *ω*(*g*(*x*)) if *g*(*x*) = *o*(*f*(*x*)) [31].

**Proof**

When *N* is sufficiently large, the maximum value of an exponential random variable, max⁡_*n*_{|*h*_*rm*,*n*_|^2^}, scales as ln⁡*N* with high probability [9]. By applying the aforementioned argument to (13), we have

(20) $$\begin{array}{c} R_{2}\approx E\left[\frac{1}{2}\log\left(1+{\tilde{\text{SNR}}}_{2,\max}\right)\right], \end{array}$$

where

(21) $$\begin{array}{c} {\tilde{\text{SNR}}}_{2,\max}=\frac{{\bar{\gamma }}_{br}{\left|h_{br}\right|}^{2}{\bar{\gamma }}_{rm}\ln N}{{\bar{\gamma }}_{br}{\left|h_{br}\right|}^{2}+{\bar{\gamma }}_{rm}\ln N+1}. \end{array}$$

We first consider the case where ${\bar{\gamma }}_{br}=o(\ln N)$. Then, it follows that

(22) SNR~2,max⁡=γ¯brhbr2γ¯rmγ¯brhbr2/ln⁡⁡N+γ¯rm+1/ln⁡⁡N≈γ¯brhbr2,

where the approximation comes from the fact that the random variables ${\bar{\gamma }}_{br}|h_{br}{|}^{2}/\ln(N)$ and 1/ln⁡⁡*N* tend to zero with high probability under the condition ${\bar{\gamma }}_{br}=o(\ln N)$. Thus, (20) can be rewritten as

(23) R2≈E12log⁡1+γ¯brhbr2=12∫0∞log⁡1+xpγbrxdx.

Using (15) in (23) and taking the integral with respect to *x* simply yield (19a). Now let us turn to the case where ${\bar{\gamma }}_{br}=\Omega (\ln N)$. Similarly as in the first case, we then obtain

(24) $$\begin{array}{c} {\tilde{\text{SNR}}}_{2,\max}\approx \left\{\begin{array}{cc} {\bar{\gamma }}_{rm}\ln N & \text{if}\;{\bar{\gamma }}_{br}=\omega \left(\ln N\right)\\ \beta {\bar{\gamma }}_{rm}\ln N & \text{if}\;{\bar{\gamma }}_{br}=C_{0}\ln N, \end{array}\right. \end{array}$$

for some constant *C*_0_ > 0, where *β* = |*h*_*br*_|^2^/(|*h*_*br*_|^2^ + *C*_1_) for some constant *C*_1_ > 0. It thus follows that 0 < *β* ≤ 1. Here, if ${\bar{\gamma }}_{br}=\omega (\ln N)$, then *C*_1_ = 0. Otherwise (i.e., if ${\bar{\gamma }}_{br}=C_{0}\ln N$), *C*_1_ > 0. This results in (19b), which completes the proof of this theorem.

From the result of Theorem 4, the following interesting observations are made. It is seen that if the average SNR of the BS-RS link, ${\bar{\gamma }}_{br}$, is relatively much smaller than ln⁡*N*, that is, ${\bar{\gamma }}_{br}\ll \ln N$, then the multiuser diversity gain is not fully exploited. It means that increasing the number of MSs, *N*, beyond a certain value is not beneficial in terms of performance on the achievable rate. Specifically, when ${\bar{\gamma }}_{br}$ is fixed (and thus does not scale with *N*), the rate *R*_2_ is bounded by a constant even for large *N*. Hence, we may conclude that it may not be desirable for all the MSs to feed back their instantaneous SNR of the BS-RS-MS link to the BS. On the other hand, if ${\bar{\gamma }}_{br}$ scales relatively faster than ln⁡*N*, that is, ${\bar{\gamma }}_{br}\gg \ln N$, then we can fully obtain the multiuser diversity gain as in (19b). In this case, as the number of MSs reporting their instantaneous SNR to the BS increases, the higher average achievable rate can be obtained.

## 5. Numerical Evaluation

In this section, to verify our analytical results in Section 4, we perform numerical evaluation via computer simulations, which show the average achievable rates for some transmission strategies under consideration. We then demonstrate the advantage of our scheduling method.

The analytic results are based on closed-form expressions for the average achievable rates of one- and two-hop transmissions, shown in (8), (14a), and (14b), respectively. On the other hand, the simulation results are obtained by using Monte-Carlo simulations for the average achievable rates of one- and two-hop transmissions, shown in (7) and (13), respectively. In our simulation, the channel coefficients in (1) and (2) are generated 3 × 10^3^ times for each system parameter.

**Figure 2:**
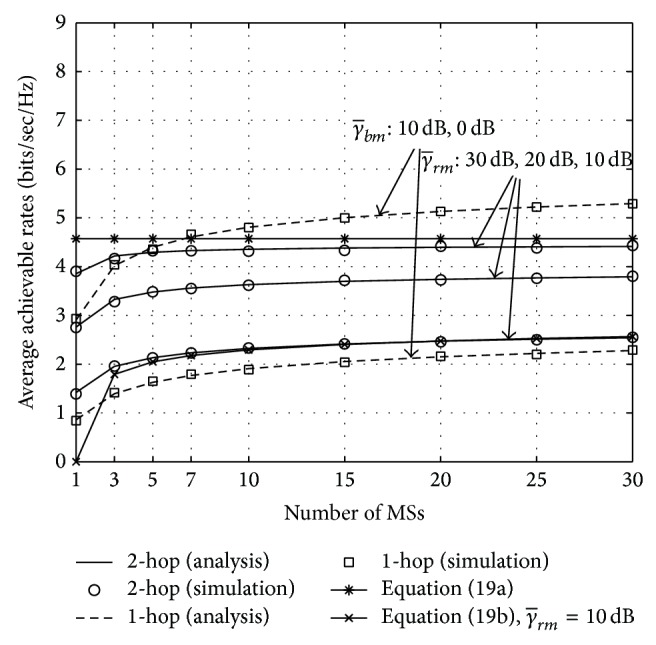
The average achievable rates versus the number of MSs, *N*, when ${\bar{\gamma }}_{br}=30\;$dB.

Figure 2 shows the average achievable rates versus the number of MSs, *N*, when we perform either one- or two-hop transmission with multiuser scheduling. We assume that *N* = 1,…, 30. The system performance is examined according to various average SNRs—it is assumed that the average SNR of the BS-RS link, ${\bar{\gamma }}_{br}$, is 30 dB, the average SNRs of the BS-MS link, ${\bar{\gamma }}_{bm}$, are 0 and 10 dB, and the average SNRs of the RS-MS link, ${\bar{\gamma }}_{rm}$, are 10, 20, and 30 dB. It is worth noting that the achievable rate for the proposed two-step multiuser scheduling in Section 3 follows the outermost boundary of two curves for either one- or two-hop transmission with multiuser scheduling. It is first seen that when ${\bar{\gamma }}_{bm}=10\;$dB, ${\bar{\gamma }}_{rm}=30\;$dB, and *N* = 1, the average achievable rate for two-hop transmission is higher than that for one-hop transmission. However, as *N* increases, performance on the achievable rate for one-hop transmission case becomes higher owing to more multiuser diversity gain. The analytical results are also illustrated in this figure, where they almost match well with the simulation results for any average SNRs and *N*. Their asymptotic behaviors for large *N* are examined as follows: we obtain the rate 4.5 (bits/s/Hz) from (19a) while the curve for ${\bar{\gamma }}_{rm}=10\;$dB is obtained from (19b) (*β* = 1 is assumed in this case). Interestingly, when *N* increases over 10 for ${\bar{\gamma }}_{rm}=30\;$dB, the average achievable rate of two-hop transmission is asymptotically upper-bounded by 4.5 (bits/s/Hz). It is further seen that the analytical result in (19b) and the simulation one for two-hop transmission with ${\bar{\gamma }}_{rm}=10\;$dB almost match for *N* ≥ 5.

**Figure 3:**
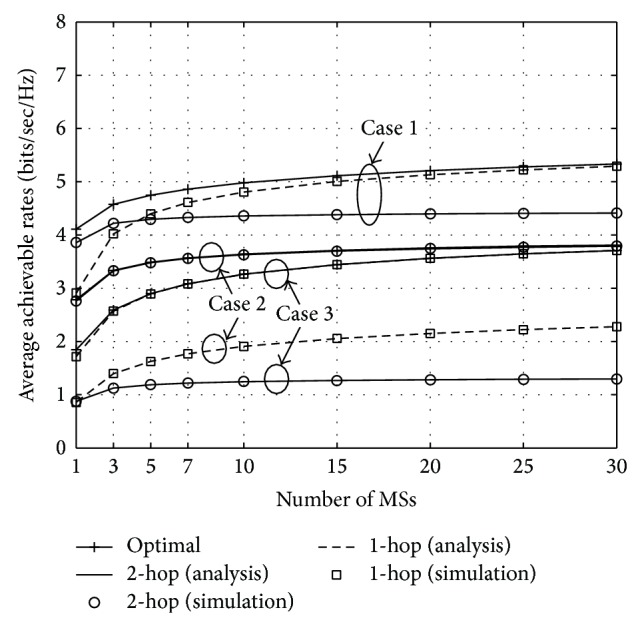
Comparison of the optimal and proposed two-step multiuser scheduling in terms of the average achievable rates for the following three cases: ${\bar{\gamma }}_{br}=30\;$dB, ${\bar{\gamma }}_{rm}=30\;$dB, and ${\bar{\gamma }}_{bm}=10\;$dB (case 1), ${\bar{\gamma }}_{br}=10\;$dB, ${\bar{\gamma }}_{rm}=10\;$dB, and ${\bar{\gamma }}_{bm}=5\;$dB (case 2), and ${\bar{\gamma }}_{br}=30\;$dB, ${\bar{\gamma }}_{rm}=20\;$dB, and ${\bar{\gamma }}_{bm}=0\;$dB (case 3).

Moreover, to see the fundamental limit of our multiuser scheduling, the comparison for the average achievable rates between the optimal and proposed two-step multiuser scheduling is performed in Figure 3. Note that, unlike the optimal scheduling such that the transmission mode is selected based on the instantaneous SNRs of all links, the proposed scheduling selects the transmission mode only using the average SNRs. For *N* = 1,…, 30, the following three cases are taken into account: (1) ${\bar{\gamma }}_{br}=30\;$dB, ${\bar{\gamma }}_{rm}=30\;$dB, and ${\bar{\gamma }}_{bm}=10\;$dB (case 1); (2) ${\bar{\gamma }}_{br}=10\;$dB, ${\bar{\gamma }}_{rm}=10\;$dB, and ${\bar{\gamma }}_{bm}=5\;$dB (case 2); and (3)  ${\bar{\gamma }}_{br}=30\;$dB, ${\bar{\gamma }}_{rm}=20\;$dB, and ${\bar{\gamma }}_{bm}=0\;$dB (case 3). For case 1, the optimal scheduling shows a slightly better performance than that of the proposed one, which is given by the outermost boundary of two curves for either one- or two-hop transmission, especially on the crossover where two curves meet. In this case, as an example, when *R*_1_ > *R*_2_, the optimal scheduling may select two-hop transmission according to some link conditions. This phenomenon occurs only for the case where there exists a crossover between two curves. However, it is easily seen that the proposed two-step scheme always outperforms either one- or two-hop transmission. On the other hand, for cases 2 and 3, the proposed two-step scheduling scheme shows nearly the same performance as that of the optimal one. In these cases, the achievable rates for the optimal scheduling are also nearly identical to those for either one- or two-hop transmission. This is because there is no crossover point between two curves of one- and two-hop transmission under the assumed link conditions. It in turn means that the proposed scheduling method works well when one transmission mode is dominant over all values of *N*. In other words, our two-step scheduling method based on efficiently reduced feedback information can be either optimal or suboptimal in terms of achievable rates, depending on link conditions (e.g., the average SNRs of the three links, denoted by ${\bar{\gamma }}_{bm}$, ${\bar{\gamma }}_{rm}$, and ${\bar{\gamma }}_{br}$).

## 6. Conclusion

The multiuser scheduling method, opportunistically selecting both the transmission mode and the desired MS, was proposed for relay-aided downlink systems using half-duplex AF relaying. The scheduling criterion, based on efficiently reduced feedback information, was designed by showing the closed-form expression for the average achievable rates and its asymptotic behaviors for large *N*. Furthermore, we analyzed the link condition such that the multiuser diversity gain is fully exploited. Finally, it was examined that the proposed algorithm has almost the same achievable rate as that of the optimal one, while its required feedback is fairly reduced by half of the optimal one.
